# Habitat-Specific Population Growth of a Farmland Bird

**DOI:** 10.1371/journal.pone.0003006

**Published:** 2008-08-20

**Authors:** Debora Arlt, Pär Forslund, Tobias Jeppsson, Tomas Pärt

**Affiliations:** Department of Ecology, Swedish University of Agricultural Sciences, Uppsala, Sweden; University of Oxford, United Kingdom

## Abstract

**Background:**

To assess population persistence of species living in heterogeneous landscapes, the effects of habitat on reproduction and survival have to be investigated.

**Methodology/Principal Findings:**

We used a matrix population model to estimate habitat-specific population growth rates for a population of northern wheatears *Oenanthe oenanthe* breeding in farmland consisting of a mosaic of distinct habitat (land use) types. Based on extensive long-term data on reproduction and survival, habitats characterised by tall field layers (spring- and autumn-sown crop fields, ungrazed grasslands) displayed negative stochastic population growth rates (log λ_s_: −0.332, −0.429, −0.168, respectively), that were markedly lower than growth rates of habitats characterised by permanently short field layers (pastures grazed by cattle or horses, and farmyards, log λ_s_: −0.056, +0.081, −0.059). Although habitats differed with respect to reproductive performance, differences in habitat-specific population growth were largely due to differences in adult and first-year survival rates, as shown by a life table response experiment (LTRE).

**Conclusions/Significance:**

Our results show that estimation of survival rates is important for realistic assessments of habitat quality. Results also indicate that grazed grasslands and farmyards may act as source habitats, whereas crop fields and ungrazed grasslands with tall field layers may act as sink habitats. We suggest that the strong decline of northern wheatears in Swedish farmland may be linked to the corresponding observed loss of high quality breeding habitat, i.e. grazed semi-natural grasslands.

## Introduction

The quality of breeding habitats strongly influences individual fitness by affecting reproductive and survival rates. In heterogeneous environments spatially varying habitat quality leads to habitat-specific demography affecting the structure and dynamics of populations by determining habitat-specific settlement patterns, population regulation, and population persistence [1]–[5]. To understand the population dynamic processes of species inhabiting heterogeneous environments it is therefore important to identify habitats of different quality. High quality habitats, where reproduction exceeds mortality may act as sources, whereas low quality habitats, where populations are maintained by net immigration, may act as sinks [2]. Both the amount of high and low quality habitat is important for population persistence as the former directly contributes to population growth, whereas the latter may stabilise the dynamics of populations by temporally hosting a surplus of individuals that otherwise would die or permanently emigrate (i.e. the buffer effect [6]–[8]).

Variation in habitat quality has often been estimated by habitat-specific densities or reproduction. However, density has been shown to not always reflect habitat quality, e.g. because of the influence of social dominance [9]–[11] or non-ideal habitat selection [12]. Estimates of single fitness components may also be misleading because they may compensate each other (e.g. low reproduction may be compensated for by high survival [13], and because it is often not clear how much they contribute to population growth [14]–[15]. Thus, habitat quality may be most closely reflected by a compound estimate of fitness of individuals as e.g. habitat-specific population growth rate [16, but see 17].

One group of organisms that have experienced large scale habitat changes during the last decades is farmland birds. During the same time many farmland birds have been declining in many European countries [18]–[19]. These declines have been attributed mainly to decreases in the amount and quality of habitat caused by agricultural intensification [18], [20], or abandonment [21]–[22]. Although much work has been done to identify the causes of population declines of farmland birds, including studies investigating whether the suggested causes actually affect demographic rates [e.g. 23–26], no study has investigated the effects of different habitat (land use) types on population growth. But data on habitat-specific growth rates are needed if we want to identify the habitat types crucial to population persistence and better predict the population dynamic consequences of landscapes changes.

Habitat-specific demography is usually investigated at the patch and population scale by contrasting two or more different habitats that vary in quality [e.g. 27–29]. For many species, however, different habitats do not occur as large and spatially uniform patches, but intersect each other to create mosaics where habitat quality varies on a smaller spatial scale, e.g. on the scale of territories [30]. When habitat quality varies at a small spatial scale habitat-specific demography and its contribution to population growth may be estimated on the scale of individual territories by linking territory-specific demography with territory habitat characteristics [e.g. 16,31]. This applies to agricultural landscapes that typically consist of a mosaic of different habitat types, and where many farmland bird species are found breeding in several of these habitat types. As farmland habitats are well-defined due to distinct land use types, farmland birds provide an ideal situation to investigate habitat-specific demography and its contribution to population growth.

The migratory northern wheatear *Oenanthe oenanthe* (hereafter wheatear) is an insectivorous, ground-foraging species mainly found in habitats consisting of bare ground or short field layers, and thus frequently inhabiting agricultural landscapes [32]. Wheatear populations have declined in many parts of the species' distribution across Europe [19]. In Sweden this decline has been about 60% between 1976 and 2001 [22], possibly due to the loss of grazed grasslands and nesting sites like stone piles and walls [33]. Here we estimated habitat-specific demographic parameters and population growth of wheatears, breeding in a farmland in southern Central Sweden, using data from a long-term population study. Restricted dispersal in combination with detailed monitoring allowed us to estimate reliable reproductive and local survival rates for this open population. In our study area wheatears occupy territories characterised by distinct land use types. Previous results show that wheatears breeding on territory sites characterised by permanently short field layers have higher reproductive success than those breeding on territories with growing (tall) field layers probably because of higher food availability and lower nest predation risk at sites with short field layers [34]–[37]. Each of these two territory classes, however, contained several land use types. Therefore, to directly link demography to land use we estimated the population growth rate of each land use type separately. Based on the previous results, we predicted population growth, on average, to be higher in land use types characterised by permanently short field layers (grazed grasslands and farmyards) than in land use types characterised by tall field layers (ungrazed grasslands, and crop fields). Furthermore, to identify potential causes of the recent population decline we examined which demographic parameters had the greatest impact on the observed differences in population growth rates among land use types.

## Methods

Our study area (60 km^2^) is a heterogeneous agricultural landscape situated southeast of Uppsala in southern Central Sweden (59°50′N, 17°50′E). In this area wheatears arrive in mid-April to mid–May and the first pairs start egg laying in early May. Wheatears incubate for about 12 days after the penultimate egg has been laid and chicks fledge from the nest at an age of about 15 days. In our study area the majority of young fledge around mid June. After fledging parental care lasts about another two weeks before the young become independent. The birds start their migration back to Africa in August [32], [35], [37]. Since 1993 all previously occupied territory sites and all sites potentially suitable for wheatears were monitored throughout the breeding season. For this study we use data from 11 years (1996–2006) for which we had territory-specific data on habitat types available. We used breeding data collected in a central and intensively studied part (40 km^2^) of the total study area, containing 149 recorded territory sites of which about 90 were occupied per year. About 97% of all breeding males and 76% of all females could be aged as either young (i.e. one year old) or old (i.e. at least two years old) based on plumage characteristics [see 35]. Male and female age of pair members were highly correlated (L-R [Likelihood-Ratio] χ^2^ = 79.44, P<0.0001, N = 820). Nest sites were abundant and nests were placed either at the ground under stones (in stone piles and stone walls) or under roof tiles of farm buildings (20%). Each year we uniquely colour-ringed nestlings from 89% of all successful nests (11% were inaccessible), as well as a proportion of adults, so that, on average, an equal proportion of 56% of breeding males and females were marked at the end of the breeding season. Territories were recorded on detailed maps (scale 1∶10 000). The location of a territory was determined by territory descriptions based on observations of the resident pair or unpaired male (<3%) made during >10 visits, excluding occasional observations of long-distance foraging or exploration movements. The locations of individual territories were relatively stable across years irrespective of territory holder, because wheatears frequently use landscape features such as prominent stones, stonewalls or fences as territory boundaries [see also 35–37].

### Habitat types

**Figure 1:**
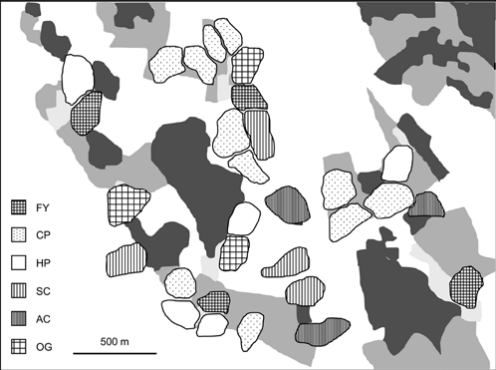
Spatial distribution of territory sites of the six different habitat types in a part of the study area. The landscape is generally composed of woodlands and forest (dark grey areas), grasslands (medium grey), built-up area (villages and farmyards; light grey), and crop fields (white). FY: farmyard, CP: cattle pasture, HP: horse pasture, SC: spring crops, AC: autumn crops, OG: other ungrazed grassland.

The landscape of our study area consists of a mosaic of grazed and ungrazed grasslands (∼10%), crop fields (∼65%), woodlands and forest (∼20%), and farmyards and other built-up area (<5%). Territory sites were spatially scattered and located in grasslands (on average 61%), crop fields (21%) and on farmyards (18%). Each year we categorized each territory site as belonging to one of the following six habitat types characterised by different land use: (1) farmyards including bare ground, mowed lawns and gardens (farmyard, FY); (2) pastures grazed by cattle, or in a few cases by sheep (cattle pasture, CP); (3) pastures grazed by horses (horse pasture, HP); (4) spring-sown crop fields (spring crops, SC; mainly oat, wheat, barley); (5) autumn-sown crop fields (autumn crops, AC; mainly wheat); (6) and other, ungrazed grasslands (other grassland, OG; grasslands mowed for silage or hay, ungrazed pastures, other unmanaged grassland habitats). The first three habitat types were generally characterised by field layers kept permanently shorter than 5 cm, whereas the latter three habitat types were characterised by field layers growing dense and tall (≥15 cm) during late incubation and nestling care (field layer height estimated by eye at four occasions during the breeding season [35], [37]). Whereas some spring crops still provided relative short and sparse field layers when early breeding pairs cared for their nestlings, autumn crops typically had reached dense and tall field layers at that time. Each territory site we assigned habitat types according to the predominant habitat type found around the nest site. When the nest site was at the border between two habitat types we assigned the habitat type with the shortest field layer height because this was the preferred foraging habitat (Arlt D & Pärt T, personal observations). This resulted in a mosaic of territory sites of the different habitat types (Fig. 1). Because habitat types could change across years for a specific territory site, we pooled data from all territories characterised by the same habitat type.

Most between-year changes of habitat types were between SC, AC and OG habitats, whereas FY, CP and HP habitats were temporally fairly stable. Wheatears (54% of males) frequently shift territory between years [38] and thus sometimes shifted between habitat types. In general, there was a net flow of adults moving from crop field habitats (SC and AC, to some extent also OG) to grazed grasslands (CP and HP; unpublished data). Of males breeding in more than one year 54% made a transition (either due to territory shifts or land use change) between habitat types at least once (unpublished data). The proportions of habitat types occupied by breeding wheatears were relatively stable across the years of study, with an average of 18% of the occupied territory sites in FY, 41% in CP, 12% in HP, 15% in SC, 6% in AC and 8% in OG habitat (unpublished data).

### Demographic rates

As we had more complete age-specific data for males all demographic rates (vital rates) were based on male data. Since reproductive performance is lower for young than for old birds [37], [39] we estimated age-specific breeding success and the number of fledged young separately for males breeding in the different habitat types (Table 1). First-year and adult survival were estimated specifically for each habitat type. Among adults we did not separate between young and old males as they had similar survival (Low M, Arlt D, Eggers S, Pärt T, unpublished manuscript). Probability of breeding was estimated separately for both adult age classes but uniformly, i.e. averaged, across all habitat types. All demographic rates were estimated using the pooled data from all 11 years.

**Table 1:** Habitat- and age-specific parameter estimates (demographic rates) and environmental variances used to model population growth.

	FY	HP	CP	SC	AC	OG
*demographic rates* 1						
breeding success Y	0.783	0.639	0.673	0.681	0.482	0.538
	(60)	(36)	(128)	(63)	(27)	(26)
breeding success O	0.860	0.741	0.755	0.742	0.838	0.613
	(107)	(83)	(289)	(80)	(31)	(49)
no. of fledglings^2^ Y	4.89	4.42	5.18	4.44	3.69	5.19
	(26)	(15)	(62)	(30)	(10)	(9)
no. of fledglings^2^ O	5.82	5.29	5.30	4.62	4.79	5.15
	(44)	(42)	(188)	(41)	(18)	(25)
first-year survival	0.256	0.316	0.279	0.296	0.168	0.212
	(187)	(164)	(1116)	(160)	(97)	(156)
adult survival	0.477	0.620	0.510	0.337	0.412	0.568
	(74)	(60)	(248)	(48)	(27)	(39)
*env. variances* 3						
breeding success Y	0.0052	0.0070	0.0067	0.0066	0.0076	0.0075
breeding success O	0.0053	0.0085	0.0082	0.0084	0.0060	0.0105
no. of fledglings Y	0.0369	0.0301	0.0415	0.0305	0.0211	0.0417
no. of fledglings O	0.0038	0.0031	0.0032	0.0024	0.0026	0.0030
first-year survival	0.0022	0.0025	0.0023	0.0024	0.0016	0.0019
adult survival	0.0005	0.0004	0.0005	0.0004	0.0004	0.0005

Parameter estimates are based on the age class of the male member of a pair, Y: young (i.e. one year old) males, O: old males. FY: farmyard, CP: cattle pasture, HP: horse pasture, SC: spring crops, AC: autumn crops, OG: other grassland. Numbers in parentheses refer to sample sizes.

1 Uniform demographic rates, i.e. estimated across all habitat types, were probability of breeding for young (0.645) and old males (0.995; see Methods for details).

2 The number of fledglings shown here refers to the total number produced by a pair.

3 Estimated environmental variance component (estimation method differs between demographic rates; see Methods for details). Data were insufficient to calculate environmental variance for probability of breeding.

#### Breeding success

Breeding success was recorded as successful or failed. A breeding attempt was defined to be successful when we observed fledglings or heard intense warning calls of the parents after fledging [35], [37]. Nest failures, on average 30%, were mostly due to predation [35]. Data on breeding success were missing when the nest had not been visited at or after the time of fledging (about 12% of all breeding attempts).

#### Fledgling production

The number of fledged young was determined by the number of nestlings ringed (when 5–8 days old) minus the number of dead chicks found in the nest after fledging. Partial nest predation is extremely rare (<1% of all successful attempts with observations of fledglings). Data on the number of fledged young were missing from 28% of all successful nests due to inaccessibility or missing data on the presence of dead chicks in the nest after fledging. When pairs renested after nest failure we used reproductive output of the final breeding attempt. True second broods were rare (0–3 per year) and omitted from the estimation.

#### Local first-year survival

In our population wheatears display a high degree of philopatry, i.e. on average 18% of all marked juveniles return to breed in the study area. Since we lack data on the sex of fledglings our survival estimates assume similar survival for males and females and an equal sex-ratio. Almost all local recruits (i.e. 95%) recruited to the population within two years after birth. We therefore limited our analysis to the cohorts of the years 1996–2004. We estimated first-year survival for marked fledglings from successful nests originating from a restricted and most central part of our study area (8 km^2^, 83 territory sites, occupied by 45–75 pairs per year) but returning to the entire 60 km^2^ area in subsequent years, thereby minimising biases due to natal dispersal. Since we identified all breeding pairs in the 60 km^2^ area all individuals dispersing within 6 km from the outer limits of the 8 km^2^ area were detected. Wheatears in our population display restricted dispersal with a median natal dispersal distance (distance between centres of territory sites) for recruits originating from the 8 km^2^ area of 1308 m (10/90% quantile = 470/3388 m; N = 203). There was no difference in recruitment or dispersal probability of juveniles from this 8 km^2^ area with respect to birth site location (central vs. peripheral territory sites [37]). We used a standard Cormack-Jolly-Seber (CJS) live mark-recapture model without time dependency in the program MARK [40] to estimate habitat-specific survival probabilities based on resighting histories of 1880 marked fledglings. We retrieved estimates from a time-independent model φ[hab]p[.] specifying survival probability (φ) as dependent on habitat type (hab), but with constant (.) resighting probability (p). Estimates of first-year survival appeared unbiased with respect to habitat type as the proportion of recruits dispersing shorter or farther than the median natal dispersal distance (see above) did not differ between individuals originating from different habitat types (L-R χ^2^ = 2.89, N = 202, P = 0.72).

#### Local adult survival

We estimated adult male survival for birds breeding in the 40 km^2^ central area between years 1996–2004 and returning to the entire 60 km^2^ area in subsequent years. In this way all individuals dispersing within 2 km from the outer limits of the 40 km^2^ area were detected. Adult males disperse much shorter distances than juveniles (median dispersal distance between centres of territory sites occupied in two subsequent years of males shifting territory site: 384, 10/90% quantile = 163/1535 m, N = 62; based on males originating from the 8 km^2^ area; t-test, log-transformed distances: t = −9.48, DF = 259, P<0.0001). We used a multistate mark-encounter model [41] in the program MARK to estimate habitat-specific adult survival probabilities based on 564 records of 329 individual adult males with known breeding history. Multistate models allow estimating state-specific survival probabilities when individuals could be found in different states (habitat types). We retrieved survival estimates from a time-independent model S[hab]p[.]ψ [hab] specifying survival probability (S) and transition probability (ψ) as dependent on habitat type (hab), but with constant (.) resighting probability (p). Dispersal distances were not biased with respect to habitat type as the proportion of males dispersing shorter or farther than the median breeding dispersal distance did not differ between males originating from different habitat types (L-R χ^2^ = 5.79, N = 59, P = 0.33).

#### Probability of breeding

Probability of breeding for young males was estimated across all habitat types from the resighting pattern of recruits that returned to breed. Of 195 male recruits 31 returned to the study area for their first time when more than one year old (14% when two, 4% when three, 1% when four years old). These individuals were used to calculate the expected number of one year old recruits R_exp,t_ from each respective cohort (year t) as R_exp,t_ = R_obs,t+1_+R_obs,t+2_/P_t+1_+(R_obs,t+3_/P_t+2_)/P_t+1_ , where R_obs,t+n_ is the number of recruits from cohort of year t first observed in year t+n, and P_t+n_ the adult male survival rate from year t+(n−1) to year t+n. Probability of breeding for young males was estimated as PrB_Y_ = R_obs_/R_exp_ , where R_obs_ and R_exp_ are the number of observed recruits and the expected number of recruits, respectively, summed up across years. Similarly, based on records of males that were recorded in non-consecutive years, i.e. which were not recorded breeding in one year (N = 3), probability of breeding among old males was calculated as PrB_O_ = M_b_/M_exp_ , where M_b_ is the number of males observed breeding, and M_exp_ the number of males expected to be alive, both summed up across years (M_exp,t_ = M_obs,t+1_+M_obs,t+2_/P_t+1_+(M_obs,t+3_/P_t+2_)/P_t+1_ , where M_obs,t+n_ is the number of males not recorded breeding during year t but in year t+1). In total we included 593 records of marked old males breeding between years 1996–2004 and returning to the study area in subsequent years.

### Population growth rate

To estimate population growth λ in the different habitat types we used a male based matrix model with two stages (based on the two age classes, see above) and post-breeding census:
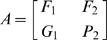
where *G_1_* was first-year survival (first-year probability of survival), P_2_ the probability of annual adult survival, and F_1_ and F_2_ fertilities of young and old males, respectively. Fertilities F_i_ were estimated from the breeding parameters (see Table 1) probability of breeding p_b,i_, probability of successful breeding p_s,i_, mean number of fledged sons per successfully breeding male f_i_ (i.e. total number of fledged young per pair divided by two, assuming an equal sex-ratio), and probability of transition to next stage G_i_ or P_i_ as F_i_ = p_b,i_×p_s,i_×f_i_×G_i_ (or P_i_). As individuals in the second stage eventually die (primarily affecting P_2_) we adjusted the model by recalculating matrix entries assuming a fixed-stage duration approach [42], where the time duration in the second stage was maximum longevity(10 years)−1.

We used habitat-specific projection matrices from which we calculated deterministic values of λ, stable stage distributions, sensitivities and elasticities [42]. To calculate long-term population growth rate in the different habitats assuming temporal environmental variance, i.e. stochastic log growth rate (log λ_s_), we used computer simulations [42]–[43] based on the six matrices. We estimated temporal environmental variance of the demographic rates by removing sampling variance from the total observed variance [43]–[44]. Small sample sizes per habitat type and year did not allow reliable estimation of habitat-specific environmental variance, and we therefore pooled data from all habitat types for each year. Hence, we assume the same temporal environmental variance in the different habitat types. For number of fledged young per successful breeding attempt we calculated environmental variance according to Morris & Doak ([43] equation 8.1). We rescaled the variance of habitat-specific demographic rates by calculating the coefficients of variation for the pooled estimates of these demographic rates and then used these coefficients of variation to calculate habitat-specific environmental variances. For binary demographic rates, i.e. survival and probability of successful breeding, we used the method of Kendall [43]–[44]. Environmental variances of the binary demographic rates were rescaled by first calculating the ratio σ^2^_e_/(p×(1−p)), where σ^2^_e_ is the temporal environmental variance as calculated above, and p×(1−p) is the maximum possible variance for a rate (where p is the overall value for the rate in the population [45]). We then used this ratio to rescale the habitat-specific environmental variances according to the habitat-specific rate. Data were insufficient to calculate temporal environmental variance for probability of breeding. We estimated Pearson correlation coefficients of annual means for demographic rates with annual estimates.

Following Morris & Doak [43] we simulated population growth by generating, for each time step (year), a set of random demographic rates (of those with annual estimates) that were correlated according to the empirical correlation matrix (Table 2). The correlations among the demographic rates generated by this method corresponded well with the empirical correlations. Each year-specific set of demographic rates was used to build a habitat- and year-specific population matrix A_t_. We started each simulation with a population at stable stage distribution according to the specific habitat type. The population vector in the following year was then estimated as n_t+1_ = A_t_×n_t_. The log growth rate log λ for each simulated time step was estimated as ln(N_t+1_/N_t_), where N is population size. The stochastic log growth rate for each habitat type log λ_s_ was calculated as the mean of log λ for all years. Each simulation was run for 10,000 time steps to yield accurate estimates of log λ_s_ and its variance.

**Table 2:** Within-year correlations of demographic rates.

	BS(O)	fled(Y)	fled(O)	surv(juv)	surv(ad)
breeding success Y [BS(Y)]	0.028	−0.338	−0.061	−0.129	0.496
breeding success O [BS(O)]		0.155	0.458	0.447	0.556
no. of fledglings Y [fled(Y)]			0.450	**0.830**	0.130
no. of fledglings O [fled(O)]				0.541	**0.685**
first-year survival [surv(juv)]					0.394

Age specific estimates for components of reproductive performance are based on the age class of the male member of a pair, Y: young (i.e. one year old), O: old. Significant correlations (P<0.05) are indicated in bold.

### Life table response experiment

To evaluate how the differences in demographic rates between our six habitat types translate into changes in λ we performed a Life Table Response Experiment (LTRE [42]). We used a fixed one-way design aimed to assess how much each demographic rate contributes to the difference in the deterministic estimate of λ between one habitat type (treatment) and another (reference habitat type). In our analysis we used pairwise comparisons assigning each habitat type in turn as the reference habitat in the comparisons. We decomposed the difference in λ between habitat types into contributions from the demographic rates as:
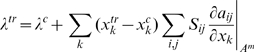
where λ^tr^ is the estimated deterministic λ of a treatment, λ^c^ is the λ for the reference habitat, x_k_ are the demographic rates, a_ij_ represents matrix elements of the habitat-specific matrices, s_ij_ is the sensitivity of matrix element a_ij_ at the mean matrix A^m^ (A^m^ = [A^tr^+A^c^]/2), and 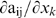 is the partial derivative of matrix element a_ij_ at the mean matrix A^m^ to changes in demographic rate x_k_. The contributions from demographic rates are calculated as an application of the chain rule and their total contribution is the sum of all contributions where this parameter is involved [42].

We estimated confidence intervals (CI) of the contributions using a bootstrap procedure that generated new estimates of all habitat-specific the demographic rates based on our empirical data. For survival rates and breeding success (binary rates) we assumed a binomial distribution. For number of fledged young we assumed a stretched beta distribution [43] and used habitat- and stage-specific means (Table 1), standard deviations, and minimum and maximum from the data pooled over all habitats and years as parameters for the distribution. Standard deviations were rescaled by calculating coefficients of variation. We generated 5000 bootstrap samples for each demographic rate. Each set of bootstrapped demographic rates was used to generate a new habitat specific projection matrix and to calculate contributions as above. From the distributions of the 5000 estimates we calculated the 95% CI for each contribution.

## Results

**Figure 2:**
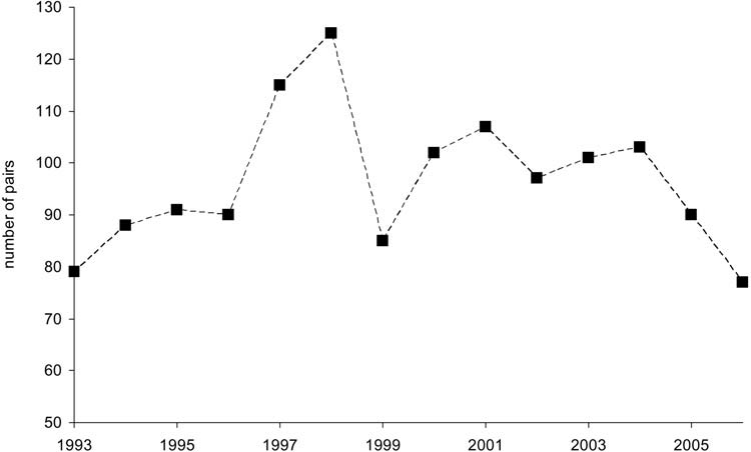
Population size of wheatears in the central study area (40 km^2^) 1993–2006.

Over a total of 14 years between 1993 and 2006 population size (number of breeding pairs) fluctuated, but showed no long-term trend (Fig. 2). Across these years most occupied territories were found in CP (41%, range = 36–47%), followed by FY (18%, 15–21%), SC (15%, 10–20%), HP (12%, 7–17%), OG (8%, 4–16%), and AC (6%, 2–16%).

### Demographic rates

The probability of successful breeding varied among habitat types for both age classes (univariate χ^2^-tests; young males: L-R χ^2^ = 9.86, N = 340, P = 0.079; old males: L-R χ^2^ = 13.38, N = 640, P = 0.019), and was, pooling young and old males, highest for FY (83.2%), followed by CP>HP>SC>AC, and lowest for OG (58.7%; Table 1 shows data for both age classes separately). Similarly, the number of fledged young from successful breeding attempts varied among habitat types (univariate ANOVA; young males: F_5,146_ = 2.81, P = 0.019; old males: F_5,352_ = 3.63, P = 0.003). Pooling young and old males, pairs fledged most young in FY (mean±SD: 5.39±1.12), followed by OG>CP>HP>SC, and least in AC (4.32±1.49; Table 1). Adult survival clearly varied between habitat types as a model with constant (habitat-independent) survival and transition probabilities S[.]p[.]ψ [.] received much lower support than the habitat-dependent model S[hab]p[.]ψ [hab] (based on model comparison using Akaike's Information Criterion corrected for effective sample size AIC_c_ [46], difference between AIC_c_ values Δ AIC_c_ = 90.4). Adult survival was highest for individuals that bred in HP (62.0%) followed by OG>CP>FY>AC and was lowest in SC (33.7%; Table 1). For first-year survival a model with constant survival probability φ[.]p[.] received similar support as the φ [hab]p[.] model (difference between AIC_c_ values Δ AIC_c_ = 1.9). First-year survival was highest in HP (31.6%) followed by SC>CP>FY>OG and was lowest in AC (21.2%; Table 1).

### Habitat-specific population growth

**Figure 3:**
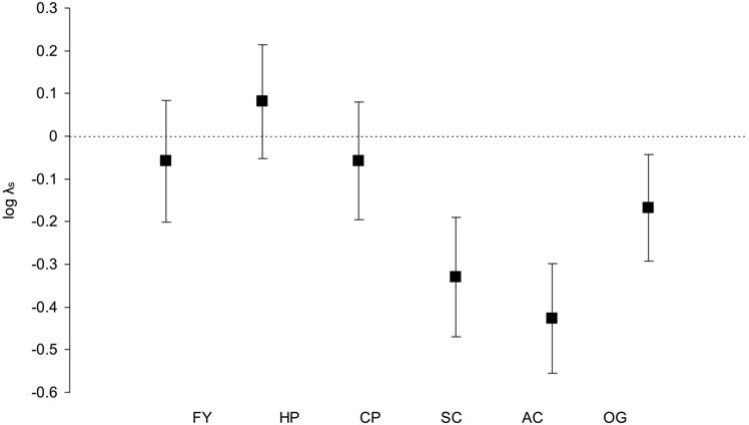
Habitat-specific stochastic population growth rates (log λ_s_) for the six habitat types. FY: farmyard, HP: horse pasture, CP: cattle pasture, SC: spring-sown crops, AC: autumn-sown crops, OG: other ungrazed grassland. Log λ_s_ was estimated from 10000 simulations of log λ. Error bars show the standard deviations of estimates of log λ resulting from temporal environmental variances.

The estimated stochastic log growth rate log λ_s_ varied greatly across the different habitat types from −0.429 in AC to +0.081 in HP (Fig. 3), these values corresponding to a 35% population decrease and a 8% increase per year, respectively (based on λ = exp[logλ_s_]). Population growth rates log λ_s_ were clearly lower in SC and AC, both habitat types being characterised by tall field layers, than in habitat types characterised by a permanently short field layer (FY, HP, and CP) (Fig. 3). The third habitat type with tall field layers, OG, had a population growth intermediate between the two types of crop field and the short field layer habitat types. All tall field layer habitat types clearly had log λ_s_<0 (Fig. 3), i.e. growth rates characteristic for declining populations. Deterministic growth rates log λ were very similar to the stochastic estimates log λ_s_ (only slightly higher, on average 0.005±0.002 SD).

### Contribution of demographic rates to habitat-specific population growth rates

**Figure 4:**
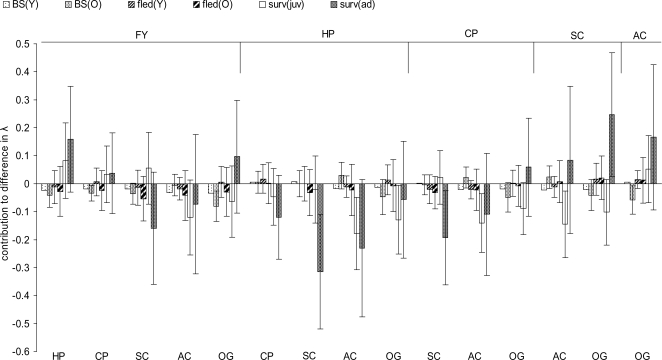
Contributions of demographic rates to differences in λ between habitat types. Contributions were estimated by LTRE using pairwise comparisons (see Methods). Error bars show 95% CI generated from 5000 bootstrap samples of the demographic rates. Labels on top refer to reference habitats and labels on bottom to treatment habitat. FY: farmyard, HP: horse pasture, CP: cattle pasture, SC: spring-sown crops, AC: autumn-sown crops, OG: other ungrazed grassland. BS(Y): breeding success of young (i.e. one year old) males, BS(O): breeding success of old males, fled(Y): number of fledglings produced by young males, fled(O): number of fledglings produced by old males, surv(juv): first-year survival, surv(ad): adult male survival.

Based on the lower level sensitivities and elasticities of the demographic rates habitat-specific λ was most sensitive to first-year and adult survival, followed by breeding success of old males and their probability of breeding (Table 3). As a result, first-year and/or adult survival were, on average, the most important demographic rates contributing to the observed differences in λ between habitat types as shown by the LTRE investigating the independent effects of the demographic rates, even though the estimated contributions displayed high variability due to sampling variance (Fig. 4). The contribution of first-year and/or adult survival to differences in λ was especially apparent (no overlap of the 95% CI with zero) in the comparisons of tall field layer habitats SC or AC with short field layer habitats HP or CP (Fig. 4). Differences in λ between habitat types were not always caused by the same demographic rates. For example, among the tall field layer habitats the lower λ in SC as compared to short field layer habitats (FY, HP and CP), was mainly due to lower adult survival, whereas the lower λ in AC was due to a lower first-year survival that contributed as much as or more than adult survival to the differences in λ (Fig. 4). Similarly, breeding success of old males, the third most important demographic rate, contributed to the differences in λ between habitat types in some comparisons (e.g. lower λ in OG as compared to short field layer habitats FY or CP; Fig. 4). All other demographic rates describing reproduction made no substantial contributions to differences of λ between habitat types (Fig. 4).

**Table 3:** Estimated projection matrix elements, and lower level sensitivities and elasticities from the habitat-specific matrices.

	FY	HP	CP	SC	AC	OG
*matrix element estimates*						
F_1_	0.316	0.288	0.314	0.289	0.096	0.191
F_2_	1.182	1.202	1.009	0.571	0.818	0.885
G_1_	0.256	0.316	0.279	0.296	0.168	0.212
P_2_	0.474	0.616	0.507	0.335	0.409	0.564
*lower level sensitivities*						
breeding success Y	0.173	0.193	0.200	0.182	0.086	0.152
breeding success O	0.318	0.376	0.310	0.178	0.226	0.335
no. of fledglings Y	0.055	0.056	0.052	0.056	0.022	0.0315
no. of fledglings O	0.094	0.105	0.088	0.057	0.079	0.080
first-year survival	1.593	1.454	1.546	1.482	1.310	1.450
adult survival	1.145	1.020	1.029	0.965	1.031	0.933
prob. breeding Y	0.210	0.191	0.209	0.192	0.064	0.127
prob. breeding O	0.275	0.280	0.235	0.133	0.190	0.206
*lower level elasticities*						
breeding success Y	0.142	0.113	0.142	0.171	0.063	0.096
breeding success O	0.288	0.256	0.246	0.183	0.289	0.242
no. of fledglings Y	0.142	0.113	0.142	0.171	0.063	0.096
no. of fledglings O	0.288	0.256	0.246	0.183	0.289	0.242
first-year survival	0.429	0.421	0.454	0.607	0.335	0.361
adult survival	0.575	0.581	0.553	0.449	0.649	0.624
prob. breeding Y	0.142	0.113	0.142	0.171	0.063	0.096
prob. breeding O	0.288	0.256	0.246	0.182	0.289	0.242

Projection matrix elements, and lower level sensitivities and elasticities were calculated according to Caswell [42]. F_1_: fertility of young (i.e. one year old) males; F_2_: fertility of old males, G_1_: first-year survival, P_2_: adult survival, FY: farmyard, CP: cattle pasture, HP: horse pasture, SC: spring crops, AC: autumn crops, OG: other ungrazed grassland, Y: young males, O: old males.

Contributions of different demographic rates to habitat differences in λ were not always in the same direction, and did partly compensate each other. For example, despite higher first-year survival and breeding success in SC as compared to OG, low adult survival resulted in a lower estimate of λ in SC. In some other comparisons juvenile and adult survival (e.g. SC vs. OG, CP vs. OG) or survival and breeding success (e.g. FY vs. HP) made opposing contributions (Fig. 4).

### Observed and expected stage distribution per habitat

The average proportion of young males observed breeding in the study area was 38%. The proportion of young males was greater in SC and AC compared to the other habitat types, but significantly so only in comparison to CP (SC: 44.5%, 95% CI = 36.9–52.4, N = 155; AC: 47.6%, 35.8–59.7, N = 63; CP: 36.7%, 26.5–35.2, N = 430; 95% CI calculated according to Newcombe & Altman [47]). In comparison with the expected proportion of young males calculated from the deterministic stable stage distribution for each habitat type the observed proportion was slightly lower in CP (30.7% observed vs. 36.1% expected, χ^2^ test, L-R χ^2^ = 5.52, P = 0.019), whereas it was greater in AC (47.6% observed vs. 28.0% expected, L-R χ^2^ = 10.92, P = 0.001) and OG (33.7% observed [N = 83] vs. 24.6% expected, L-R χ^2^ = 3.48, P = 0.062; for all other habitats P>0.29).

## Discussion

Population growth rates were higher in habitat types characterised by permanently short field layers than in those with tall field layers, suggesting marked differences in quality between these habitats. Although the estimated population growth rates varied due to temporal environmental variance, habitat types with tall field layers (spring crop, autumn crop, other grassland) had stochastic growth rates of log λ_s_ significantly lower than zero, i.e. growth rates characterising declining populations (Fig. 3). Habitat-specific differences in population growth rates thus broadly corroborated our expectations based on differences in reproduction between territories with short and tall field layers [35]–[37]. Using distinct habitat types, however, enabled us to obtain a more detailed picture with respect to different land use types, and in combination with the life table response experiment we could identify the demographic variables critical for the observed habitat-specific differences in population growth rates.

In contrast to many other studies, our results are based on modelling male demography. However, our main results of habitat-specific demography would not be altered when modelling female demography in this socially monogamous species (unpublished data). A possible exception would be a lower population growth rate in ‘other grassland’ habitat as a result of a lower adult female survival rate (unpublished data).

Variation in demographic rates between habitats largely followed patterns of habitat structure, i.e. field layer height. Breeding success was lower in habitat types where field layers were dense and tall during the nest stage, mainly due to more frequent nest predation [35]–[36]. The reduced number of fledglings in the habitats with tall and dense field layers was likely caused by lower food availability [34]–[36] because wheatears, like many other ground-foraging farmland bird species, display lower foraging efficiency and thus avoid foraging in such field layers [33], [49]. Data on parental feeding behaviour (Low M, Arlt D, Eggers S, Pärt T, unpublished manuscript) show that pairs breeding in crop fields return with smaller load sizes to their nestlings than those breeding in short field layer habitats. At the same time, these pairs markedly increased their feeding effort due to increased flight distances to nearest short field layer patches (Low M, Arlt D, Eggers S, Pärt T, unpublished manuscript). Thus, reduced first-year and adult survival rates could also be linked to reduced food availability in tall field layers.

Habitat-specific differences in population growth rate (especially the difference between spring or autumn crops and the pasture habitats) were mainly explained by variation in first-year and/or adult survival as revealed by our life table response experiment. This strong effect of first-year and adult survival rate is due to the large differences in survival rates between habitats and their relatively large sensitivities and elasticities (Table 3). There were also relative large differences in breeding success between habitats, but breeding success had lower sensitivities and elasticities. Although habitats clearly varied with respect to the number of fledglings produced per successful breeding attempt, this variation did not contribute much to the observed differences in habitat-specific differences in population growth rates. Also other studies have shown that adult and juvenile (i.e. first-year) survival rates contribute most to population growth rate even in relatively short lived species [48], [50]–[52]. Clearly, individual survival rates may have crucial effects on habitat-specific population growth, and need to be included in realistic assessments of habitat quality.

A common problem is that survival rates are the most difficult demographic rates to obtain because of the permanent emigration (dispersal) from a finite study area [53]. Dispersal distributions are typically characterised by a flat but long tail of long-distance dispersers. In an exceptionally large study area using data on natal dispersal of migratory tree swallows *Tachycineta bicolor* Winkler et al. [54] estimated that a study area of 10 km extent from the natal site would have missed 11% of the dispersing birds. We tried to minimize the influence of dispersal by only estimating survival rates for individuals originating from central parts of the whole study area, so that birds dispersing moderate distances were detected. But since we could not detect long-distance dispersers (i.e. permanent emigrants), our survival rates, and hence population growth rates, are most likely underestimated. Importantly, the underestimation of survival rates did not appear to be biased with respect to habitat types, as natal and breeding dispersal patterns did not differ between habitats (see Methods). Furthermore, habitat-dependent long-distance dispersal seems unlikely in this mosaic landscape where different habitat types occur in close proximity to each other (Fig. 1). Therefore, estimated habitat-specific differences in local survival are most likely caused by true differences in mortality. This is corroborated by our data showing that adult survival of birds breeding in crop fields is reduced due to increased parental effort (see above). The fact that birds breeding poor habitats actually experience increased parental effort also argues against the possibility that higher male survival in short field layer habitats is a result of a higher proportion of males with a previous breeding history in poor habitats with low reproductive effort. Habitat-specific survival may also be influenced by the phenotypic quality of individuals, which on average may be lower in spring and autumn crop habitats as is suggested by the higher proportion of young males occupying these habitats. However, habitat, as classified by short and tall field layers, had a strong effect on reproduction in a previous study controlling for individual phenotypic effects [36], suggesting strong habitat effects per se on demography. To summarise, the *relative* differences in population growth rates between the habitat types are unlikely to be affected by the underestimation of survival rates and reflect true differences.

Some habitats had stochastic log growth rate rates close to zero (i.e. characterising stable populations; farmyard, horse and cattle pasture), suggesting, given the underestimation of population growth rates, that these habitats could act as source habitats. On the contrary, spring and autumn crop habitats had marked negative growth rates. The relatively poor quality of these two habitat types seemed to be due to different causes. Whereas, in comparison to short field layer habitats, the low population growth rate in spring crop was mostly due to low adult survival, the low growth rate in autumn crop was rather due to low first-year survival in combination with low adult survival, potentially reflecting the low availability of invertebrate food when feeding nestlings in this more dense and tall habitat. Also ungrazed grassland habitat had a negative growth rate, which, however, seemed more caused by the relatively low breeding success caused by a high risk of nest predation (the most important nest predators, i.e. weasels *Mustela nivalis*, stoats *M. erminea* and adders *Vipera berus,* largely prefer hunting in tall field layers [55]). Based on their low population growth rates the crop fields seem to function as sink habitats. Under the assumption of adaptive habitat selection it may seem surprising that we find individuals in habitats with such low growth rates (corresponding to a 35% and 28% population decrease in autumn and spring crop habitat, respectively). At least in autumn crop habitats we observed a higher proportion of young males than was expected from the stable stage distribution for this habitat type, which may indicate immigration of individuals from other habitat types. Individuals may be forced into poor habitats when high quality habitats are saturated [6], [56]. However, our previous studies show that some wheatears actually select breeding sites in poor habitats despite good ones are available (i.e. non-ideal habitat selection [37]).

Wheatear populations have declined strongly in Swedish farmland (see Introduction). During the same time period, the area of semi-natural dry pastures (i.e. pastures with stones, boulders and bare rock, unsuitable for ploughing or mowing and with a continuous grazing regime for centuries and therefore permanently short field layers) has decreased by about 30% since the 1950's [57]. Most of our grazed pastures (i.e. the potential source habitat) were of this semi-natural type with abundant nesting sites for wheatears. Furthermore, the number of farms (and farmyards) has decreased due to either amalgamation into larger units in intensively farmed regions, or extensification and abandonment of small-scale farming in forest-dominated regions [22]. Such forest-dominated landscapes with small-scale farming had previously dense populations of wheatears [58]. Our results on habitat-specific population growth in combination with the overall observed decreases in the amount of high quality breeding habitat for wheatears, i.e. grazed grasslands and farmyards, therefore suggest that loss of high quality habitat is a major factor for the observed decline in population numbers of wheatears in Sweden. This is in line with the general suggestion that loss of habitat patches with short and sparse vegetation due to agricultural intensification may be a major threat to many populations of ground-foraging farmland birds [20], [49].

Habitat-specific demography is often interpreted in terms of habitat quality. Most studies, however, only use estimates of reproduction, e.g. breeding success or production of young, and assume that habitat-differences in these demographic parameters will be reflected in local population growth rates, but if survival is not positively correlated to reproduction [see e.g. 59] this assumption may be not true. Our study of a short-lived species showed that rankings of habitats based on breeding success or number of fledglings are not necessarily consistent with the ranking based on habitat-specific population growth rates, i.e. a compound estimate integrating reproduction and survival. Moreover, different demographic variables partly made opposing contributions compensating each other, emphasising the need to consider several demographic rates and a compound estimate of fitness to realistically assess habitat quality. In our case adult survival followed by first-year survival were the most critical demographic rates contributing to the differences between habitats in population growth. Generally, survival appears to have a strong effect on population growth rates of many bird species [50]. Thus, estimating and understanding survival rates are central for understanding the causes of population declines.
